# Supplementary material for: Variant to function mapping at single-cell resolution through network propagation
Source: bioRxiv. 2022 Jan 24:2022.01.23.477426. Preprint. [Version 1] doi: 10.1101/2022.01.23.477426 (PMC8811900; doi:10.1101/2022.01.23.477426)

Supplementary Fig. 1

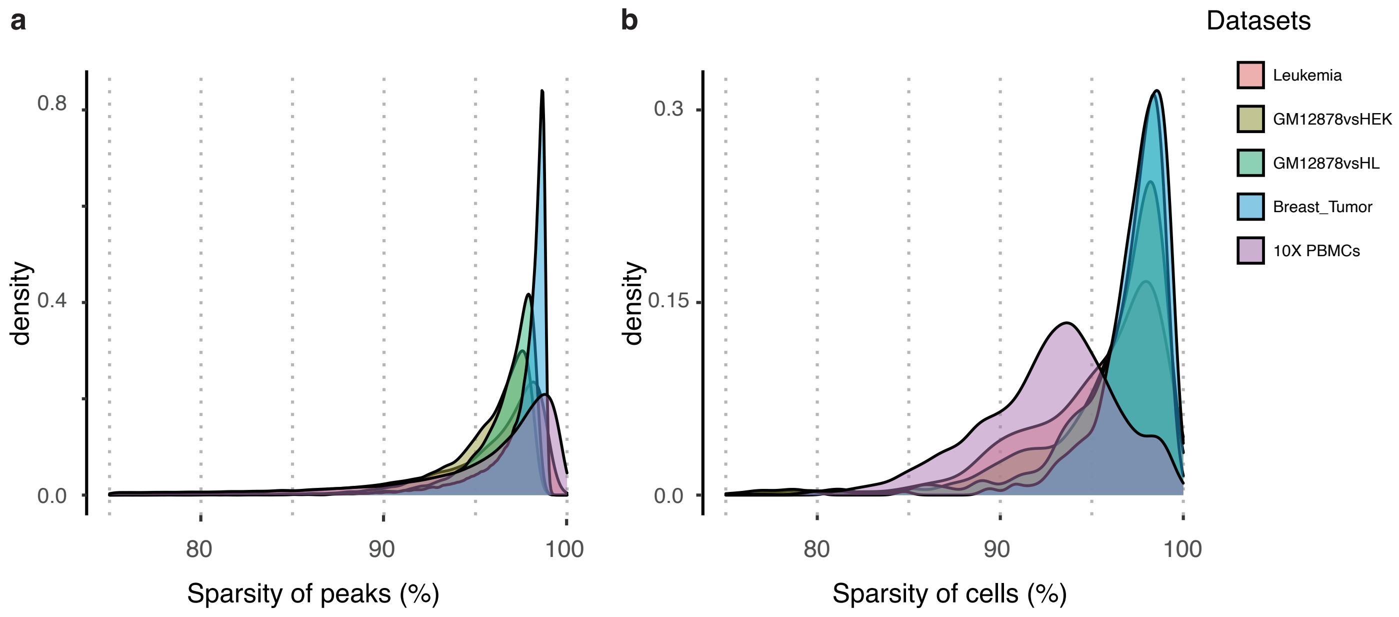

Supplementary Fig. 2

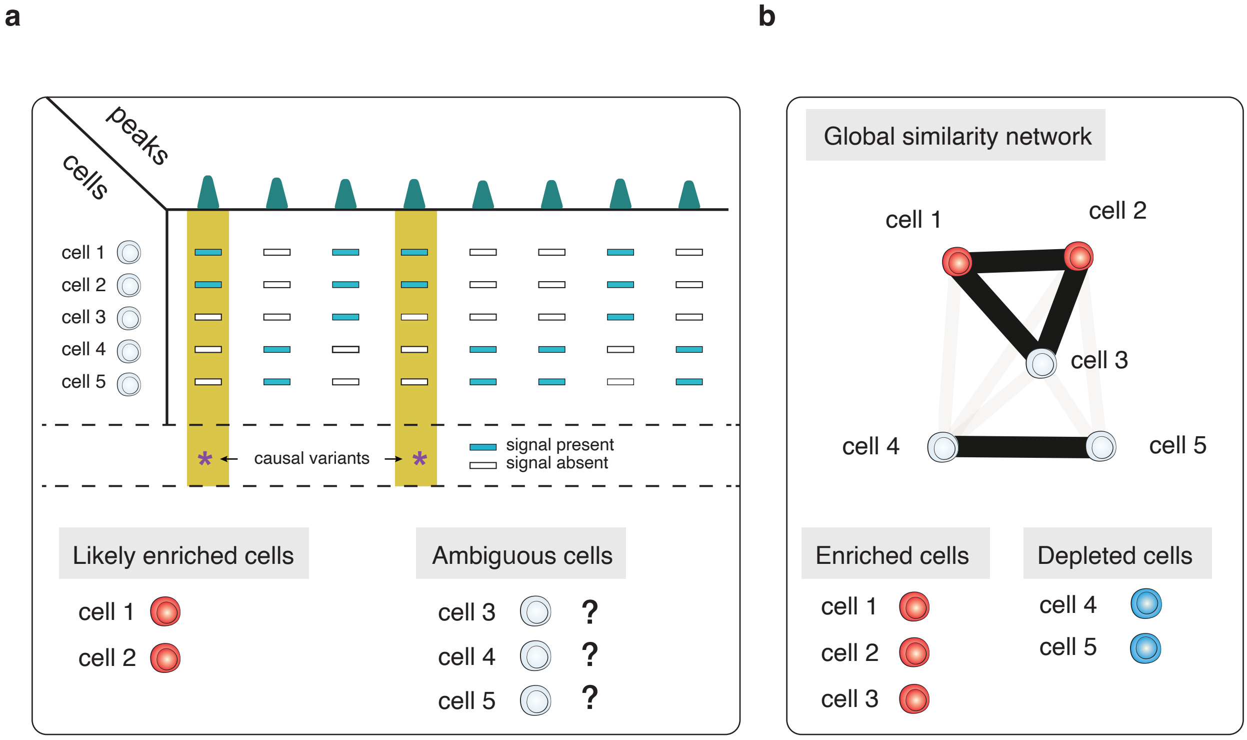

Supplementary Fig. 3

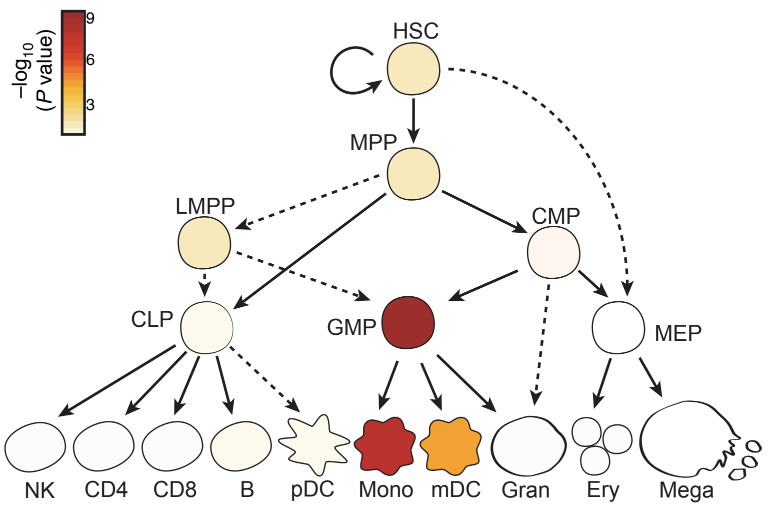

Supplementary Fig. 4

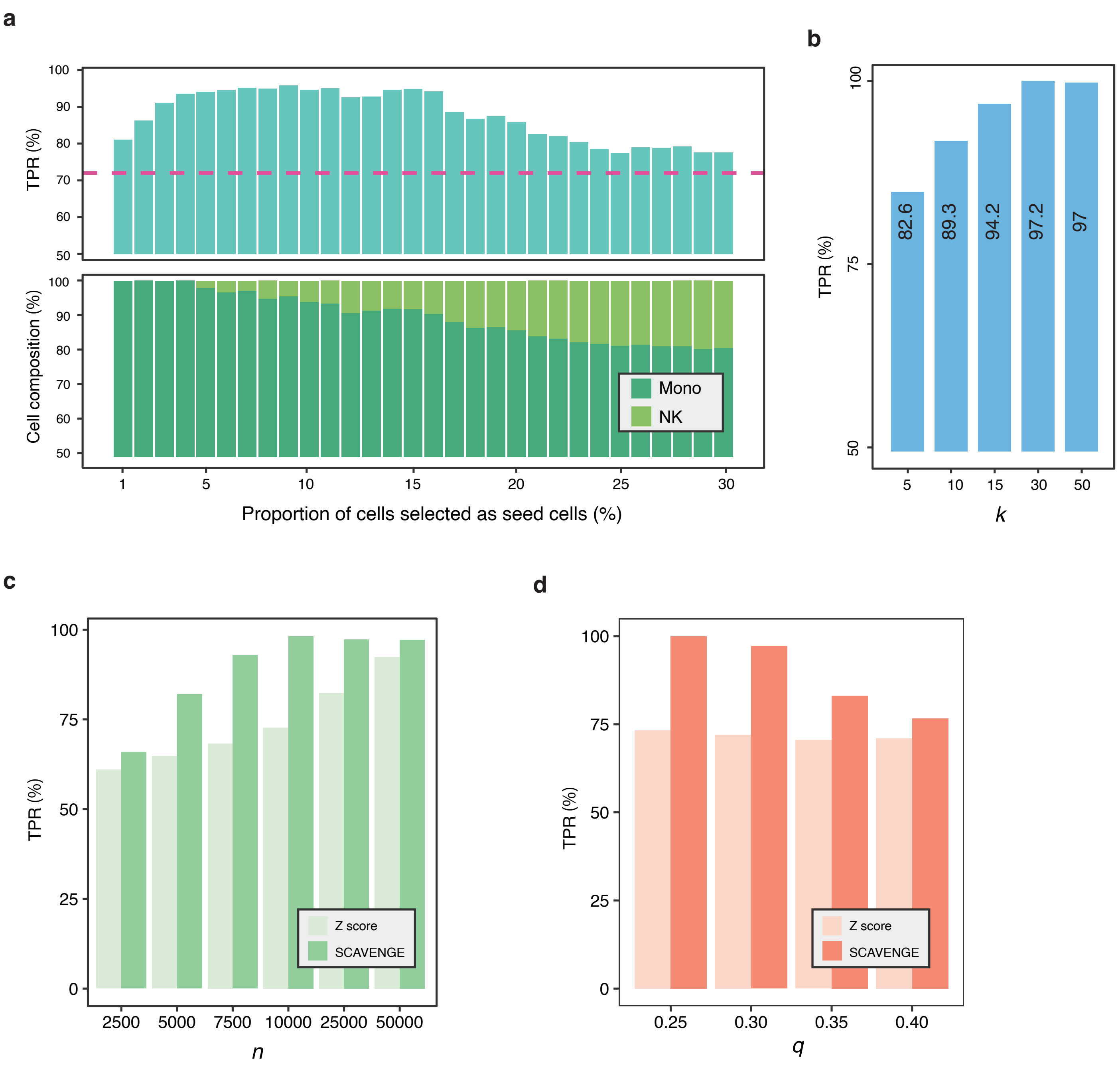

Supplementary Fig. 5

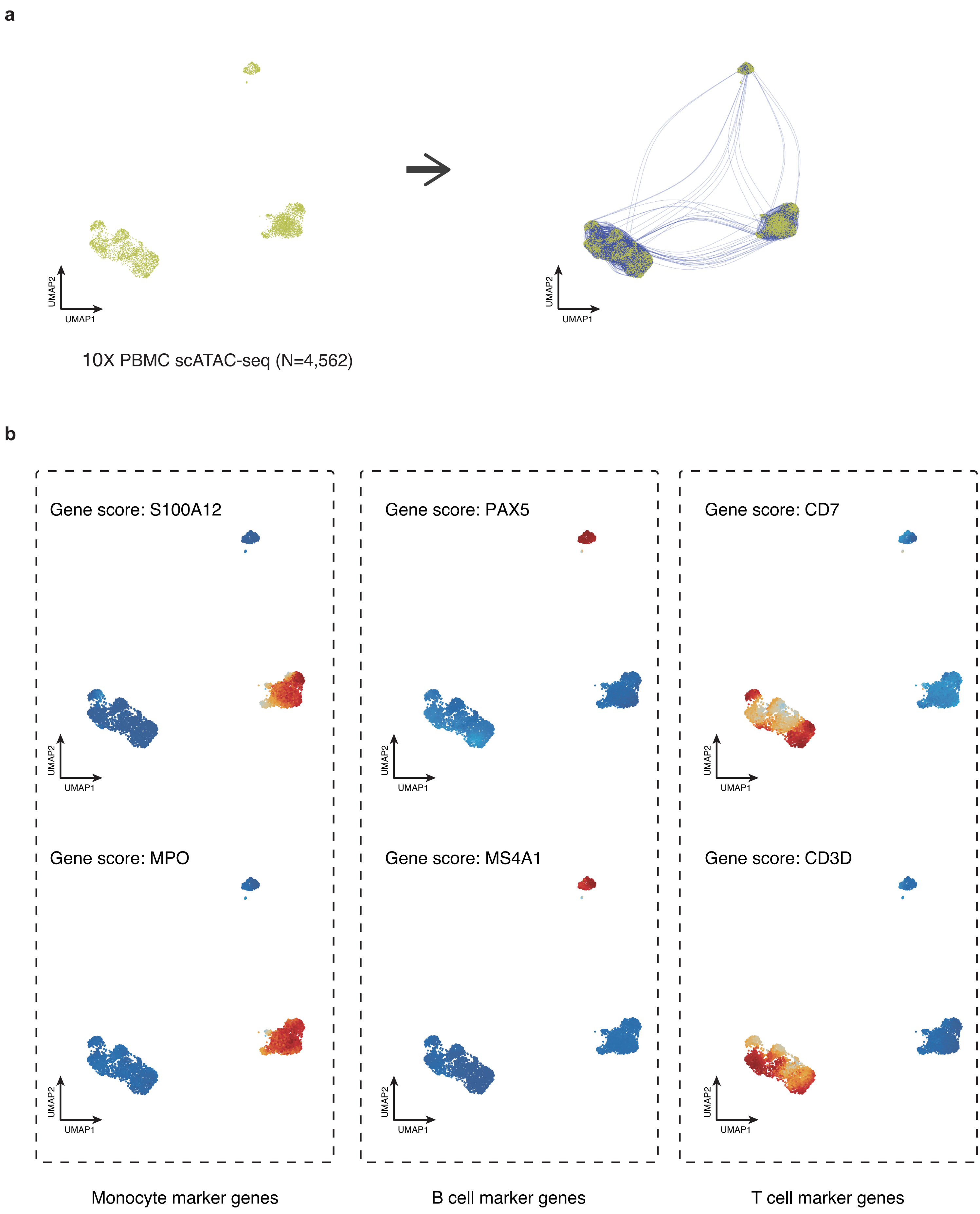

Supplementary Fig. 6

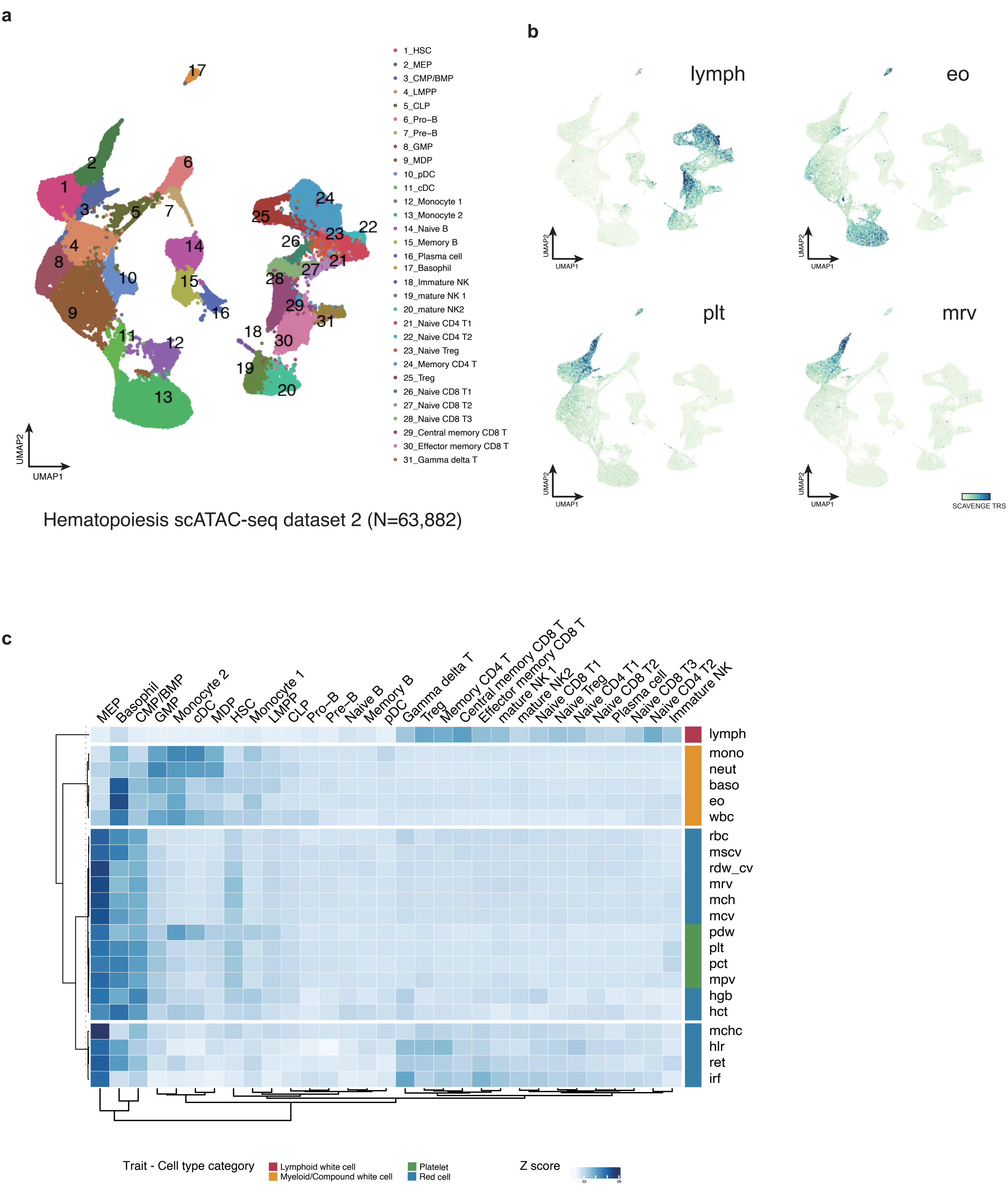

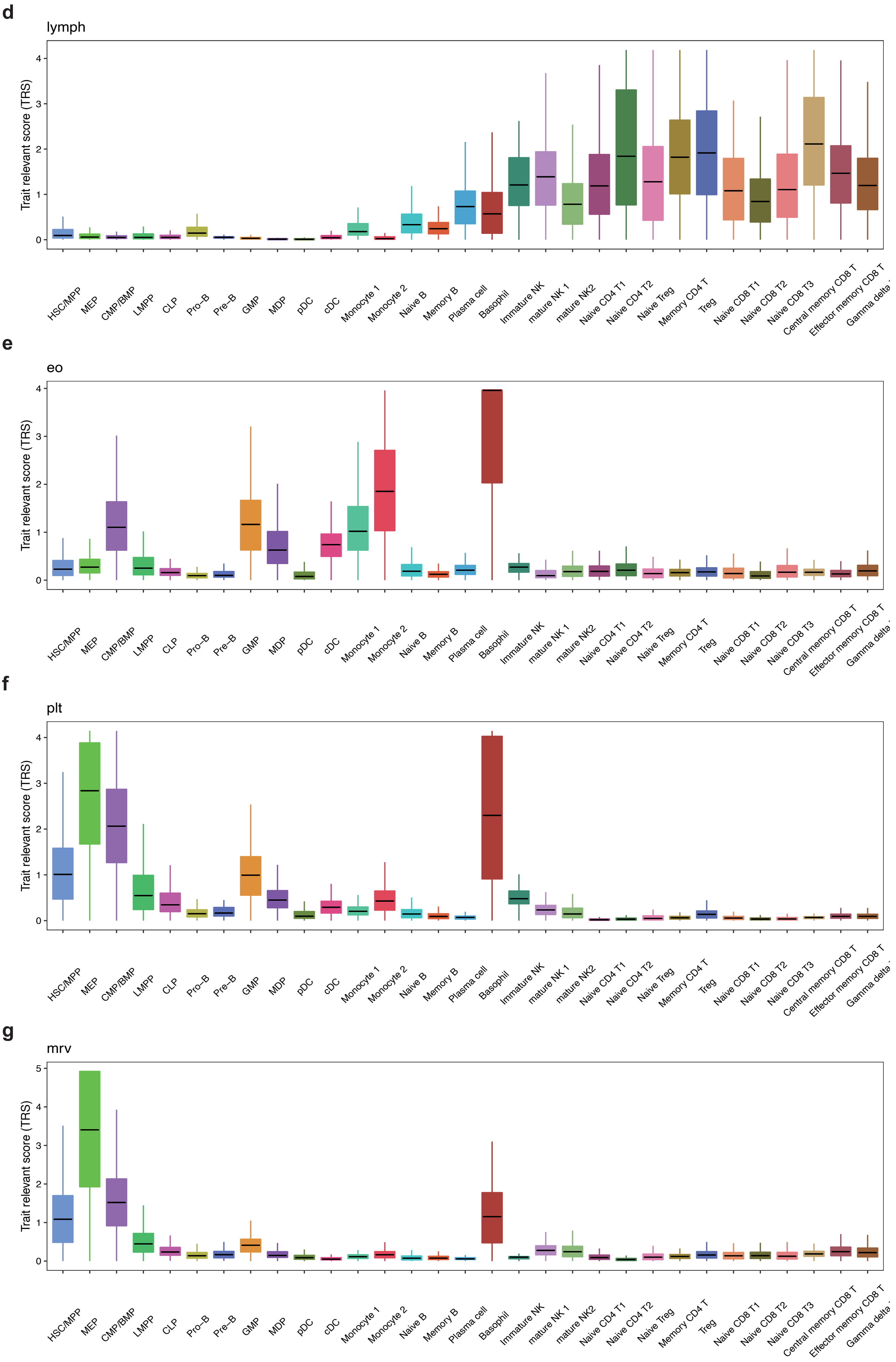
d
lymph
e
eo
f
plt
g
mrv
Trait relevant score (TRS)
0
1
2
3
4
5
HSC/MPP
MEP
CMP/BMP
LMPP
CLP
Pro–B
Pre–B
GMP
MDP
pDC
cDC
Monocyte 1
Monocyte 2
Naive B
Memory B
Plasma cell
Basophil
Immature NK
mature NK 1
mature NK2
Naive CD4 T1
Naive CD4 T2
Naive Treg
Memory CD4 T
Treg
Naive CD8 T1
Naive CD8 T2
Naive CD8 T3
Central memory CD8 T
Effector memory CD8 T
Gamma delta

Supplementary Fig. 7

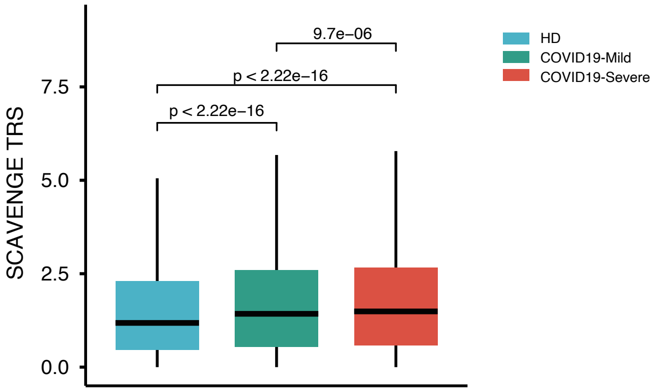

Supplementary Fig. 8

a

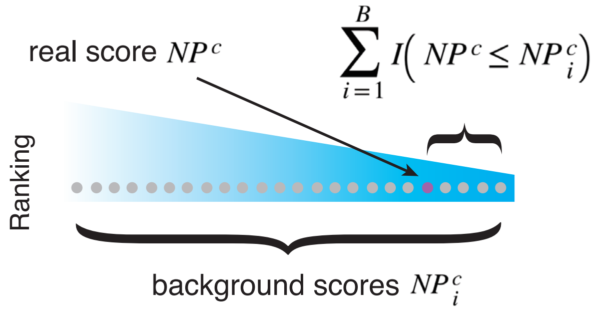

Empirical *P* value:

$$p_e^c = \frac{1 + \sum_{i=1}^{B} I\left(NP^c \leq NP_i^c\right)}{1 + B}$$

b

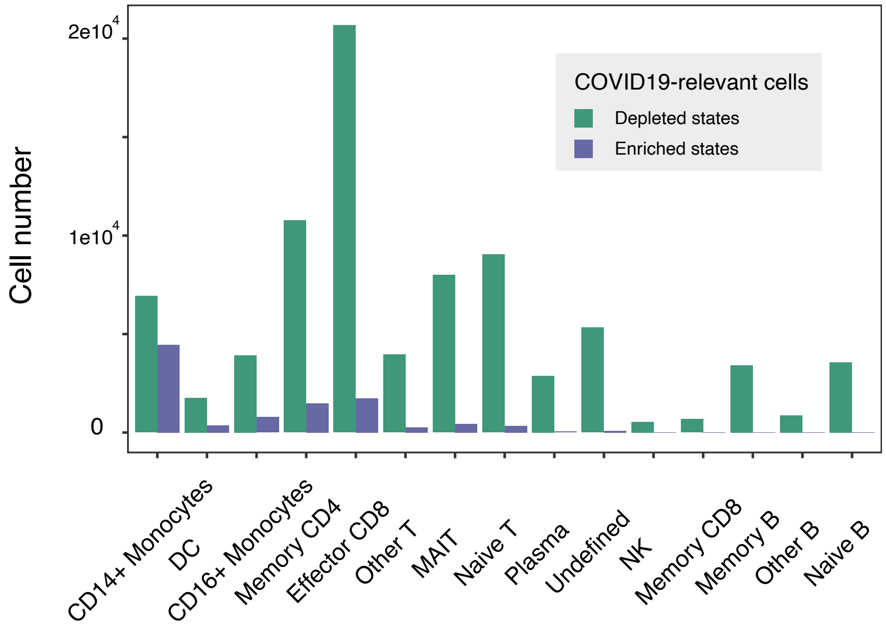

Supplementary Fig. 9

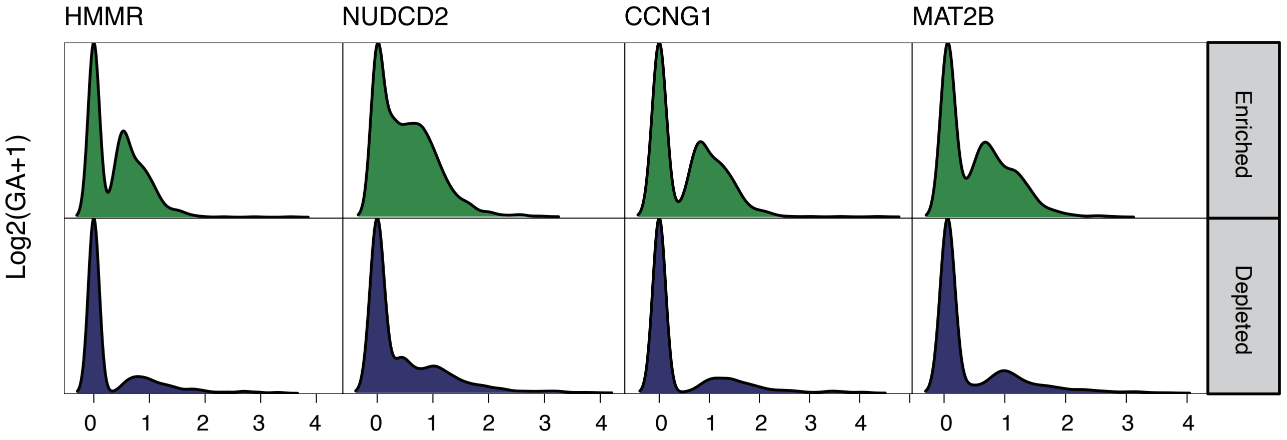

Supplementary Fig. 10

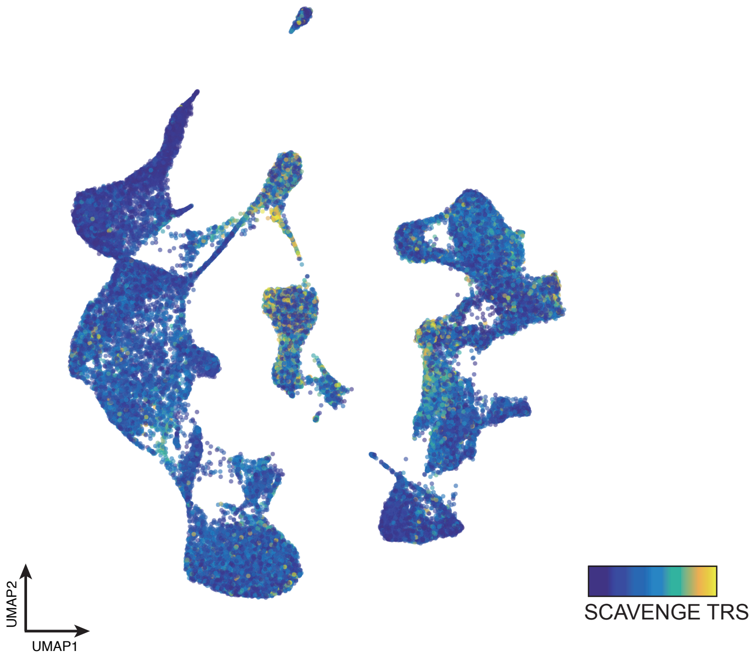

Hematopoiesis scATAC-seq dataset 2 (N=63,882)

Supplementary Fig. 11

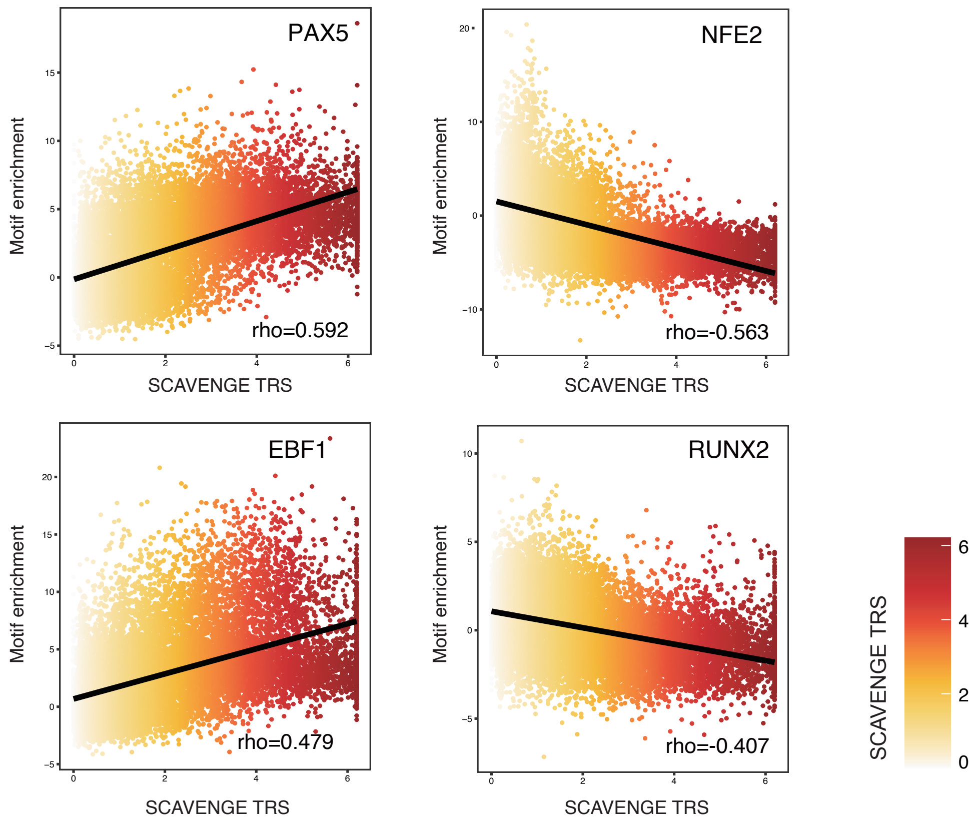

Supplement: Supplement 1 — Supplementary Fig. 1: The extensively high sparsity in scATAC-seq data. The density plots show that high sparsity commonly exists across curated scATAC-seq data. The sparsity in scATAC-seq data is characterized using the peak-by-cell matrix from five different datasets. a, The sparsity of peaks is defined as the proportion of cells that show no signal (zero-valued) for a given peak. b, The sparsity of cells is defined as the proportion of peaks that show no signal for a given cell. The 10X PBMC scATAC-seq dataset is used in the following SCAVENGE analysis. Supplementary Fig. 2: Challenges for identification of trait/phenotype-relevant cells using colocalization-based approaches and the network-based solution. To investigate the causal cell type/state that is relevant to a genetic trait, the most commonly used strategy is co-localization of epigenetic signals that occur in regulatory elements (peaks) and risk variants. However, this approach is unfortunately uninformative for a majority of cells when applied to scATAC-seq profiles. Given the noise and sparse nature of scATAC-seq data, absence of signals are extensive across cells and regulatory peaks, which can not be distinguished between technical or biological causes. Therefore, only a few cells demonstrate reliable phenotypic relevance (a). While global high-dimensional features of individual single cells are sufficient to represent the underlying cell identities or states, which enables the relationships among such cells to be readily inferred. We reason that the real relevant cell populations can be revealed and recovered by building a search engine for cell-to-cell networks that enable discovery of similar cells with the same phenotype (b). Supplementary Fig. 3: Enrichments of monocyte count associated genetic variants in bulk hematopoietic ATAC-seq data. The enrichment scores are obtained by using g-chromVAR for the trait of monocyte count with bulk ATAC-seq profiles across 16 hematopoietic cell types. Supple [file media-1.pdf]
