# Supplementary material for: Variant to function mapping at single-cell resolution through network propagation
Source: bioRxiv. 2022 Jan 24:2022.01.23.477426. Preprint. [Version 1] doi: 10.1101/2022.01.23.477426 (PMC8811900; doi:10.1101/2022.01.23.477426)
Supplement: 1 [file NIHPP2022.01.23.477426v1-supplement-1.pdf]

bioRxiv preprint doi: https://doi.org/10.1101/2022.01.23.477426; this version posted January 24, 2022. The copyright holder for this preprint (which was not certified by peer review) is the author/funder, who has granted bioRxiv a license to display the preprint in perpetuity. It is made available under aCC-BY 4.0 International license.

SUPPLEMENTARY TABLES

Supplementary Table 1: SCAVENGE analysis of monocyte count with 10X PBMC scATAC-seq dataset.

Supplementary Table 2: SCAVENGE analysis of 22 blood cell traits with Hematopoiesis scATAC-seq dataset.

Supplementary Table 3: SCAVENGE analysis of 22 blood cell traits with Hematopoiesis scATAC-seq 2 dataset.

Supplementary Table 4: Fine-mapped variants of COVID19 severity trait.

Supplementary Table 5: SCAVENGE analysis of COVID19 severity with COVID-19 PBMC scATAC-seq dataset.

Supplementary Table 6: Fine-mapped variants of the ALL risk trait.

Supplementary Table 7: SCAVENGE analysis of ALL risk predispositions with Hematopoiesis scATAC-seq 2 dataset.
